# Lymphovenous anastomosis for the treatment of persistent congenital chylothorax in a low-birth-weight infant: A case report

**DOI:** 10.1097/MD.0000000000017575

**Published:** 2019-10-25

**Authors:** Kenji Hayashida, Sho Yamakawa, Eri Shirakami

**Affiliations:** Division of Plastic and Reconstructive Surgery, Shimane University Faculty of Medicine, Izumo, Shimane, Japan.

**Keywords:** chylothorax, infant, lymphedema, lymphovenous anastomosis

## Abstract

**Rationale:**

Chylothorax remains a poorly understood phenomenon, and no optimal treatment or guidelines have been established. This is the first report of treating congenital chylothorax and lymphedema in a low-birth-weight infant by lymphovenous anastomosis (LVA).

**Patient concerns:**

We report a case of successful LVA for persistent congenital chylothorax and lymphedema resistant to other conservative therapies.

**Diagnosis:**

The diagnosis of chylothorax was confirmed by the predominance of lymphocytes in the pleural fluid draining from the chest tube. In addition, the infant developed oliguria and generalized lymphedema.

**Interventions:**

LVA under local anesthesia combined with light sedation was performed at his medial thighs and left upper arm.

**Outcomes:**

Although his subcutaneous edema markedly improved, the decrease in chest tube drainage was gradual. No additional treatment was required.

**Lessons:**

LVA is of considerable value as a surgical treatment option in the setting of persistent congenital chylothorax and lymphedema, because LVA is a less invasive procedure.

## 1 Introduction

Primary chylothorax in pediatric patients is a rare condition defined as an abnormal accumulation of lymphatic fluid or congenital anomaly of lymphatic vessels within the pleural space. The lymphatic system is a delicate plexus of thin-walled vessels that drain lymph and chyle from interstitial spaces into the venous circulation. Several studies reported the incidence of persistent congenital chylothorax to range from 1 in 10,000 to 15,000 pregnancies.^[1–3]^

Chylothorax remains a poorly understood phenomenon, and no optimal treatment or guidelines have been established. Traditional postnatal management includes tube thoracostomy in addition to some combination of high medium-chain triglyceride (MCT) enteral formula, defatted breast milk, total parenteral nutrition (TPN), and/or somatostatin analogs.^[4]^ Fetal interventions, including pleurodesis with OK-432 or surgical therapies, are generally reserved for refractory cases.^[5]^ Thoracic duct ligation has been reported, but its results are not predictable and do not offer long-lasting improvement.^[6]^

Weissler et al^[7]^ reported a novel procedure of lymphovenous bypass of the terminal portion of the thoracic duct that offered a chance of cure to patients lacking other effective therapeutic options after cardiac surgery. However, the method has a risk of continuous postoperative leakage of lymph from the surgical site and requires general anesthesia. On the contrary, a surgical technique to treat very small vessels has recently been established (super-microsurgery: anastomosis of vessels 0.5–1.0 mm in diameter), enabling lymphovenous anastomosis (LVA) for the treatment of lymphedema under local anesthesia. LVA using the super-microsurgery procedure for lymphedema is becoming popular due to its effectiveness and minimal invasiveness.^[8–10]^ We report the youngest case of successful LVA for persistent congenital chylothorax and lymphedema resistant to other conservative therapies.

## 2 Case report

The mother was referred to our hospital because of fetal hydrops with suspicion of congenital chylothorax. Fetal therapeutic thoracocentesis was performed in our obstetrics department at 29 weeks of gestation. However, the draining and thoraco-amniotic shunting were not effective. Emergency cesarean section was carried out because of amniorrhexis at 30 weeks and 3 days of gestation. The male infant weighted 1706 g at birth, and the Apgar score was 1 at 1 minute, 1 at 5 minutes, and 4 at 10 minutes. The infant had severe respiratory distress and was admitted to the neonatal intensive care unit for immediate mechanical ventilation. Initial chest radiography demonstrated severe bilateral pleural effusions. The diagnosis of chylothorax was confirmed by the predominance of lymphocytes (white blood cell count, 4214 cells/mm^3^; lymphocytes, 87.0%) in the pleural fluid draining from the chest tube. He was fed with TPN, but the pleural effusion increased. Combined bilateral chest tube drainage ranged from 150 to 200 mL/d. In addition, the infant developed oliguria and generalized lymphedema. Although octreotide infusion and steroid administration were initiated, no significant decrease in chest tube drainage was noted.

**Figure 1:**
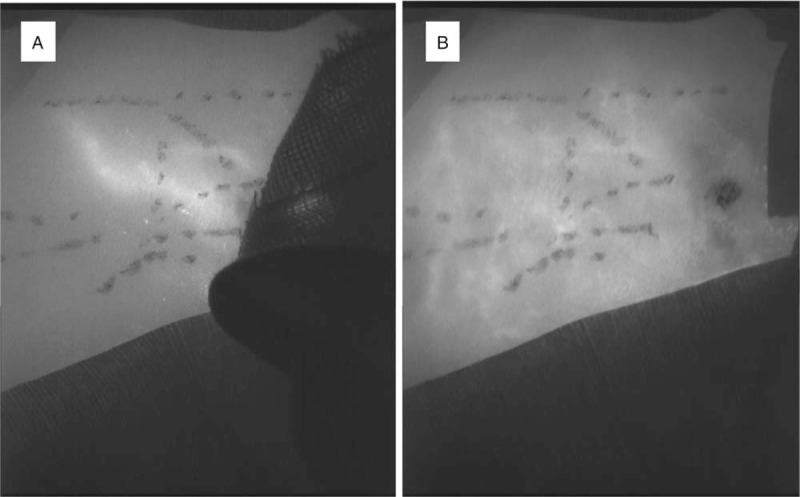
Characteristic ICG lymphography findings at his left medial thigh. Microscopic ICG lymphography pattern changed from splash to stardust. (A) A splash pattern was observed immediately after ICG injection. (B) The pattern changed to a stardust pattern within a few minutes. ICG = indocyanine green.

His body weight decreased to 1533 g on postnatal day 38. However, general lymphedema and pleural effusion flow remained unchanged. The infant was referred to our division for the treatment of lymphedema. After obtaining informed consent from his parents, we performed indocyanine green (ICG) lymphography to determine the severity of lymphedema and locate the lymphatic vessels. Indocyanine green (0.5% Diagno-green, 0.2 ml; Daiichi Pharmaceutical, Tokyo, Japan) was injected subcutaneously at the medial knee and upper arm. Vivid dynamic images of superficial lymphatic flow on magnified ICG lymphography were obtained right after the injection. Also, a splash pattern and a stardust pattern were observed in the regions (Fig. 1). As these findings indicated mild dermal backflow and the existence of lymphatic collecting vessels, we decided to perform LVA.

**Figure 2:**
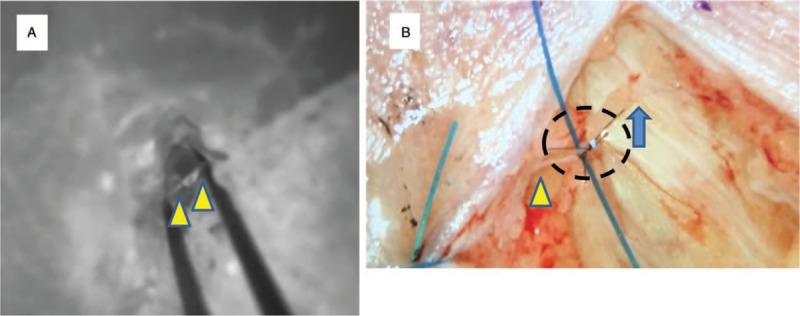
Lymphovenous anastomosis (LVA) with guidance of intraoperative microscopic ICG lymphography. (A) Intraoperative microscopic ICG enhanced a lymphatic vessel (arrowhead). (B) After completion of LVA. Circle indicates the anastomosis site. Lymph fluid flowed into a venule (arrow). ICG = indocyanine green.

Lymphovenous anastomosis under local anesthesia combined with light sedation was performed at his medial thighs and left upper arm to avoid interference between operative fields and medical devices. Incision sites were decided based on preoperative ICG lymphography using an operating microscope with the illumination system along the subcutaneous veins detected by a portable vein illumination device. After infiltration of anesthesia with 1% lidocaine and 1:100,000 epinephrine, a 1 cm-long skin incision was made. The adipose layer was dissected seeking for lymphatic vessels under the guidance of intraoperative microscopic ICG lymphography using the microscope. As ICG-enhanced lymphatic vessels were visualized under the microscope before being thoroughly dissected, we were able to dissect easily and directly toward the enhanced lymphatic vessel. After identification of the lymphatic channels and veins appropriate for anastomoses, the vessels were transected. An intravascular stent was subsequently inserted into the vein. As the vein size was approximately 0.2 mm, 7-0 size blue monofilament nylon was used as a stent. LVA using 11-0 size black monofilament nylon sutures and a hemi-intravascular stenting method was carried out.^[11]^ The lymphatic vessel was smaller than the vein and had very thin walls. However, we were able to perform three end-to-end anastomoses in total for approximately 2 hours without adverse events (Fig. 2). Patency of the anastomosis was confirmed by visualization of lymphatic fluid flow from the lymphatics to the veins using ICG lymphography.

After the operation, lymphedema around the anastomosis site decreased gradually. Before LVA, medical staffs were unable to bend his elbows because of severe lymphedema, but they were able to 2 days after LVA. Postoperatively, his thighs and left upper arm decreased in size compared with their preoperative size (left upper arm: 9.0–8.0 cm, left thigh: 13–11.5 cm, right thigh: 13–11.5 cm). As his trunk also decreased in size (30.0–27.5 cm), he was able to undergo heart surgery for patent ductus arteriosus on postnatal day 56. Although his subcutaneous edema markedly improved, the decrease in chest tube drainage was gradual. No additional treatment was required. As of postnatal day 120, the infant is alive and being fed by TPN.

Informed written consent was obtained from the patient's parents for publication of this case report and accompanying images.

## 3 Discussion

There is no previous report on the effects of LVA for congenital chylothorax and lymphedema in a low-birth-weight infant. Congenital chylothorax has previously been treated nonoperatively, and the treatment algorithms were reported by Beghetti et al.^[12]^ Their report suggests that when 1 week of MCT milk feeding and 3 weeks of TPN are ineffective at reducing the pleural chylous effusion, surgical options should be considered. Pleurodesis with OK-432 is also effective for pleural adhesion and stopping chylous leaks.^[5]^ One of the advantages of OK-432 for congenital chylothorax is its rapid effects. However, fever lasting for 2 to 4 days and local inflammatory reaction lasting for 3 to 7 days are its reported side effects.^[13]^ In addition, the long-term prognosis of pleural adhesions resulting from OK-432 exposure is unknown.

The recent development of the super-microsurgical technique has improved the ease and accuracy of LVA as a surgical procedure for lymphedema.^[8]^ Several reports have described the efficacy of LVA for primary lymphedema. Demirtas et al^[14]^ stated that LVA was similarly efficacious for both primary and secondary lymphedema. Hara et al^[15]^ stated that LVA was effective in patients who were aged 11 years or older at the time of lymphedema onset. Moreover, through continued surgical innovation, micro-surgeons remain uniquely equipped to provide novel treatment strategies for lymphatic anomalies when noninvasive methods fail. Weissler et al^[7]^ reported the use of LVA to manage refractory chylothoraces in infants after cardiac surgery. LVA provided definitive closure of refractory chyle leaks and restored normal lymphatic drainage, facilitated return to enteral feeding, and achieved liberation from mechanical ventilation. Although limited to 2 cases, the use of LVA to manage recalcitrant chylothoraces warrants consideration as a surgical treatment option for this life-threatening condition. Taghinia et al^[16]^ reported the results of LVA of the terminal thoracic duct in 14 patients with chylous leakage owing to central conducting lymphatic anomalies. They concluded that the bypass operation offered a chance of improvement or cure with relatively minimal risk for patients with refractory chyle leak secondary to central conducting lymphatic anomalies.

This is the first report of treating congenital chylothorax in a low-birth-weight infant by LVA. Based on our experience, the postoperative course after LVA was better than expected, suggesting LVA therapy as an alternative treatment option. LVA should be attempted before the injection of OK-432 because LVA is less invasive. LVA can be performed under local anesthesia like our case. Local anesthesia is a safe and effective method to perform surgery in patients unable to receive general anesthesia.^[17,18]^ It may not be necessary to wait 3 weeks to perform LVA if TPN is not effective at reducing chylous effusion and general lymphedema.

## 4 Conclusions

Lymphovenous anastomosis is of considerable value as a surgical treatment option in the setting of congenital chylothorax and lymphedema. Further prospective investigations with a longer follow-up period are needed.

## Author contributions

**Data curation:** Sho Yamakawa, Eri Shirakami.

**Writing – original draft:** Kenji Hayashida.
